# Methylation Status of SP1 Sites within miR-23a-27a-24-2 Promoter Region Influences Laryngeal Cancer Cell Proliferation and Apoptosis

**DOI:** 10.1155/2016/2061248

**Published:** 2016-03-23

**Authors:** Ye Wang, Zhao-Xiong Zhang, Sheng Chen, Guang-Bin Qiu, Zhen-Ming Xu, Wei-Neng Fu

**Affiliations:** ^1^Department of Medical Genetics, China Medical University, Shenyang 110122, China; ^2^Department of Laboratory Medicine, No. 202 Hospital of PLA, Shenyang 110003, China; ^3^Department of Otolaryngology, No. 463 Hospital of PLA, Shenyang 110007, China

## Abstract

DNA methylation plays critical roles in regulation of microRNA expression and function. miR-23a-27a-24-2 cluster has various functions and aberrant expression of the cluster is a common event in many cancers. However, whether DNA methylation influences the cluster expression and function is not reported. Here we found a CG-rich region spanning two SP1 sites in the cluster promoter region. The SP1 sites in the cluster were demethylated and methylated in Hep2 cells and HEK293 cells, respectively. Meanwhile, the cluster was significantly upregulated and downregulated in Hep2 cells and HEK293 cells, respectively. The SP1 sites were remethylated and the cluster was significantly downregulated in Hep2 cells into which methyl donor, S-adenosyl-L-methionine, was introduced. Moreover, S-adenosyl-L-methionine significantly increased Hep2 cell viability and repressed Hep2 cell early apoptosis. We also found that construct with two SP1 sites had highest luciferase activity and SP1 specifically bound the gene cluster promoter in vitro. We conclude that demethylated SP1 sites in miR-23a-27a-24-2 cluster upregulate the cluster expression, leading to proliferation promotion and early apoptosis inhibition in laryngeal cancer cells.

## 1. Introduction

miR-23a, miR-27a, and miR-24-2 consist of miR-23a-27a-24-2 gene cluster which is highly conserved in different species. miR-23a-27a-24-2 cluster and its individual members play important roles in various biological and pathological processes such as cell development [1], proliferation [2], apoptosis [3], differentiation [4], immune response [5], and invasion and metastasis [6], respectively.

Aberrant miR-23a-27a-24-2 cluster expression is reported to be a common event in lots of cancers such as acute lymphoblastic leukemia [7], acute myeloid leukemia [8], chronic lymphocytic leukemia [9], prostate and breast cancer [10], gastric cancer [11], cholangiocarcinoma [12], hepatocellular cancer (HCC) [13], acute promyelocytic leukemia [14], and colorectal cancer [15]. Because three members in the cluster are derived from a single primary transcript, they have similar expression pattern in general. For example, the cluster has been found to be upregulated in acute lymphoblastic leukemia [7], acute myeloid leukemia [8], chronic lymphocytic leukemia [9], prostate and breast cancer [10], gastric cancer [11], cholangiocarcinoma [12], and hepatocellular cancer (HCC) [13], respectively. In several cancers, such as acute promyelocytic leukemia (APL), the cluster is downregulated [14]. In colorectal cancer, however, the first two miRNAs of the cluster are overexpressed and the third is underexpressed [15]. The complex expression patterns suggest that the cluster is tissue-specific and is involved in complicated regulatory mechanism in gene expression.

However, why miR-23a-27a-24-2 cluster is aberrantly expressed is seldom reported. From genetics level, only amplification is confirmed to upregulate the cluster expression in gastric cancer cells [16]. In epigenetics, SNPs and methylation are reported to be associated with regulation of the cluster expression. For example, the polymorphisms miR-23a rs3745453, miR-27a rs895819, and rs11671784 could modulate the cluster member's expression [17–19]. In hepatocellular carcinoma, He et al. found that hypomethylation contributes to aberrant miR-23a and miR-27a expression by genome-wide methylated DNA immunoprecipitation chip and miRNA expression microarray assays [20]. miR-23a gene is hypermethylated and upregulated after demethylation in osteosarcoma cells [21]. These suggest that methylation status affects the cluster expression regulation. Unfortunately, how methylation regulates miR-23a and miR-27a expression is not reported.

In our previous study, we found that miR-23a and miR-27a are upregulated in laryngeal cancer [22, 23]. We also analyzed relationship between miR-23a-27a-24-2 cluster polymorphism rs10422126 and laryngeal cancer occurrence. However, the result showed no significant difference between them (data not shown).

In the study, we predicted CG-rich region of miR-23a-27a-24-2 cluster promoter and detected the methylation status in the region spanning two SP1 sites. We also investigated whether methylation status of SP1 sites affects the cluster expression and proliferation and apoptosis in Hep2 cells.

## 2. Materials and Methods

### 2.1. Cells and Cell Culture

Human laryngeal carcinoma cells Hep2 and human embryonic kidney cells HEK-293 were obtained from Cell Biology Institute of Shanghai, Chinese Academy of Science. Hep2 and HEK 293 cells were maintained in RPMI-1640 and Dulbecco's high glucose modified Eagle's medium (DMEM), respectively, with 10% fetal bovine serum, 100 nits/mL penicillin, and 100 *μ*g/mL streptomycin in a humidified atmosphere at 37°C with 5% CO_2_. S-Adenosyl-L-methionine (SAM) was purchased from Sigma Corporation (MO, USA).

### 2.2. Quantitative Reverse Transcription-Polymerase Chain Reaction (qRT-PCR)

To detect the expression of miR-23a/27a/24-2 cluster in Hep2 and HEK293 cell lines, total RNA was isolated using Trizol reagent (Invitrogen, Carlsbad, CA, USA) following the protocol of the manufacturer. Reverse transcription was performed using the One Step Prime Script miRNA cDNA Synthesis Kit (Takara, Dalian, China) following the manufacturer's instructions. qRT-PCR was performed using SYBR® Premix Ex Taq*™*II (Takara, Dalian, China) according to the manufacturer's instructions using 7500 Real-time RT-PCR system (Applied Biosystems, Foster City, CA, USA). PCR results were normalized to endogenous U6 and quantified in relation to the controls using the delta-delta CT method. All primers for miR-23a/27a/24-2 cluster used in the study are shown in Table 1.

### 2.3. Bisulfite Modification and Bisulfite-Specific PCR (BSP)

Hep2 cells were treated by SAM. Genomic DNAs isolated from Hep2, HEK-293, and SAM-treated Hep2 cells were used to detect methylation status of CG-rich region in miR-23a/27a/24-2 cluster promoter. Genomic DNA was then bisulfite-modified using the EZ DNA Methylation-Gold*™* kit (Zymo Research, Orange, CA, USA) according to the manufacturer's recommendation. Based on the promoter CG-rich region sequence of the cluster, bisulfite PCR primers were designed according to the online primers program “MethPrimer” (http://www.urogene.org/methprimer/). Primers used for BSP are as follows: forward 5′-TTTGTATTTTGGAGTTTGGATTTTG-3′ and reverse 5′-CCTCATTAAACCCTAAACAAACCA-3′. BSP products were then cloned into a T-vector (Takara, Japan) and transformed into JM109* E. coli* competent cells (Takara, Japan) according to the manufacturer's instructions.

### 2.4. Proliferation Assay

Hep2 cells were treated by SAM at 0.2 mM, 0.4 mM, 0.6 mM, 0.8 mM, and 1.0 mM concentrations, respectively. SAM-untreated Hep2 cells were used as controls. 3-4 × 10^4^ cells were seeded into each well of a 96-well culture plate to a final volume of 100 *μ*L. After culture for 24 h, 48 h, 72 h, and 96 h, 10 *μ*L of CCK-8 was added to each well and incubated for 1–4 h at 37°C in a 5% CO_2_ incubator. Absorbance at 450 nm was measured using a microplate reader. Growth inhibition rate was then calculated. A proliferation curve was plotted based on SAM concentration and growth inhibition rate. The subsequent concentration of SAM treatment was based on IC_50_ value.

### 2.5. Apoptosis Assay

Apoptotic cells were measured by using an Annexin-V:FITC Apoptosis Detection Kit I (BD Biosciences, San Jose, CA, USA) according to the manufacturer's protocol. Hep2 cells were incubated with 0.2 mM, 0.4 mM, 0.6 mM, 0.8 mM, and 1.0 mM SAM for 72 h, respectively. Cells were then harvested, washed twice with 1x PBS, and resuspended in 100 *μ*L of binding buffer. Cells were incubated with Annexin-V and PI at room temperature for 15 min in the dark. Apoptosis was detected by flow cytometer (FACSCalibur, Becton-Dickinson, USA). Signals Annexin-V^−^/PI^−^, Annexin-V^+^/PI^−^, and Annexin-V^+^/PI ^+^ indicate living, early, and late apoptotic cells, respectively.

### 2.6. Transient Transfection and Luciferase Assays

p450, p498, and p603 constructs containing zero, one, and two SP1 sites in the cluster, respectively, were obtained from GENECHEM (Shanghai, China). Cells seeded in 96-well plate in triplicate were transfected with different constructs by using Lipofectamine 2000*™* in accordance with the manufacturer's procedure. pRL-TK (Promega Corporation, Madison, WI, USA) was used as a normalization control. Cells were collected at 48 h after transfection and luciferase activity was measured using a dual-luciferase reporter assay kit (Promega Corporation) by Dual Luciferase Assay System (Promega, USA). Relative luciferase activity was calculated as firefly/*Renilla* luciferase ratio.

### 2.7. Electrophoretic Mobility Shift Assay (EMSA)

Nuclear extracts of Hep2 cells were prepared using a nuclear extract kit (Pierce, USA) following the manufacturer's instructions. Oligonucleotides used in EMSA were synthesized by Sangene (Beijing, China), and their sequences were as follows: SP1 wild type: 5′-CTCTGGGGGCGGGGGGGTCGG-3′ and mutant: 5′-CTCTGGAGAATAAGAGGTCGG-3′. The oligonucleotides were labeled using the biotin 3′ end DNA Labeling Kit (Pierce, USA). EMSA was performed by LightShift Chemiluminescent EMSA kit (Pierce, USA) according to the protocol provided. In brief, nuclear protein extracts were incubated with 3′-end-biotin-labeled probes in binding buffer for 20 min on ice, separated on a 6% nondenaturing polyacrylamide gel, and then transferred onto a nylon membrane and fixed by ultraviolet cross-linking. Protein-DNA complexes were visualized by streptavidin-horseradish peroxidase followed by chemiluminescent detection (Pierce, USA). For competition assays, nuclear protein extracts were incubated with a 100-fold excess of the unlabeled wild type and mutated oligonucleotide duplex competitors, respectively. For supershift reaction, anti-SP1 antibody (Abcam, USA) was incubated with nuclear extracts for 1 h at 4°C prior to the addition of the biotin-labeled DNA probes.

### 2.8. Statistical Analysis

Unless otherwise stated, each experiment was performed for a minimum of three times. Data were subjected to statistical analysis by SPSS 16.0 software and shown as mean ± standard deviation (SD). A paired samples *t*-test was used to analyze differences in miR-23a/27a/24-2 cluster expression. Results obtained from cell-based experiments were analyzed by independent samples *t*-test and one-way ANOVA. *P* < 0.05 is considered statistically significant.

## 3. Results and Discussion

### 3.1. Results

#### 3.1.1. A CG-Rich Region in miR-23a-27a-24-2 Cluster Promoter Is Hypomethylated in Hep2 Cells

**Figure 1:**
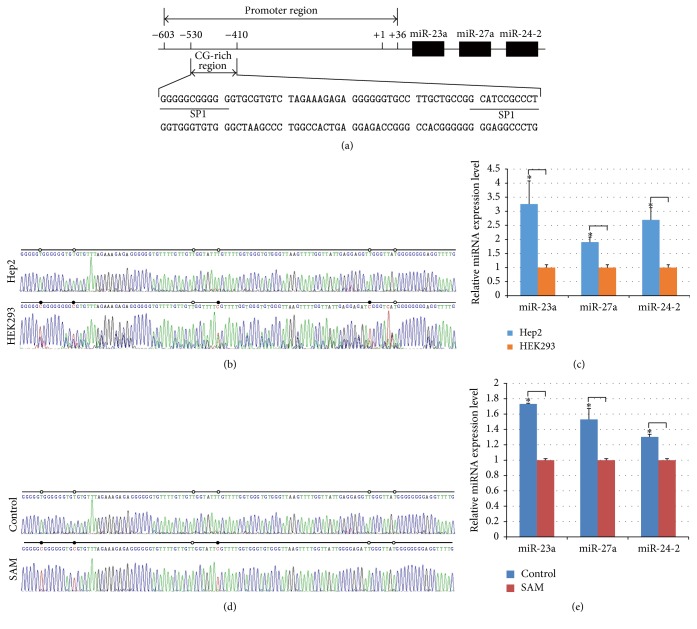
Effects of DNA methylation status of miR-23a-27a-24-2 cluster promoter CG-rich region on the cluster expression. (a) Prediction of miR-23a-27a-24-2 cluster promoter CG-rich region. CG-rich region spanning two SP1 sites is located at the cluster promoter, −530~−410. (b) DNA methylation status of the CG-rich region in Hep2 and HEK-293 cells. Cytosines of CG dinucleotides in the two SP1 sites were hypermethylated in HEK-293 cells compared to Hep2 cells. (c) Expression of miR-23a-27a-24-2 cluster in Hep2 and HEK-293 cells. Members of the cluster were upregulated in Hep2 cells compared to HEK-293 cells. (d) DNA methylation status of the CG-rich region in SAM-treated and SAM-untreated Hep2 cells. Cytosines of CG dinucleotides in the two SP1 sites were remethylated in SAM-treated Hep2 cells compared to SAM-untreated cells. (e) Expression of miR-23a-27a-24-2 cluster in SAM-treated and SAM-untreated Hep2 cells. Members of the cluster were downregulated in SAM-treated Hep2 cells compared to SAM-untreated cells. Hep2 cells were incubated with 1 mmol/L SAM. SAM-untreated cells were used as controls. *∗* indicates *P* < 0.05.

As shown in Figure 1(a), a CG-rich region with 6 CpGs overlapping two SP1 sites was found in the cluster promoter −530~−410. Bisulfite DNA sequencing results displayed that 4 cytosines in 6 CpGs (66.7%) spanning the two SP1 sites were methylated in HEK-293 cells. In Hep2 cells, none of them were methylated (Figure 1(b)), suggesting that the CG-rich region including two SP 1 sites in Hep2 cells is demethylated compared to that in HEK-293 cells.

#### 3.1.2. Hypomethylation of the CG-Rich Region Contributes to Upregulation of miR-23a-27a-24-2 Cluster in Hep2 Cells

qRT-PCR results indicated that three members of miR-23a-27a-24-2 cluster were significantly overexpressed in Hep2 cells compared to HEK-293 cells (Figure 1(c)). Furthermore, bisulfite DNA sequencing results showed that 3 cytosines in 6 CpGs (50%) including two SP1 sites were remethylated in SAM-treated Hep2 cells (Figure 1(d)), implying that SAM can alter the methylation status in the CpG-rich region. qRT-PCR result displayed that miR-23a-27a-24-2 cluster expression level was significantly lower in SAM-treated Hep2 cells than that in SAM-untreated ones (Figure 1(e)). These results suggest that hypomethylation of the CG-rich region especially two SP1 sites upregulates the cluster expression.

#### 3.1.3. Hypomethylation of the CG-Rich Region Participates in Regulation of Hep2 Cell Proliferation and Apoptosis

**Figure 2:**
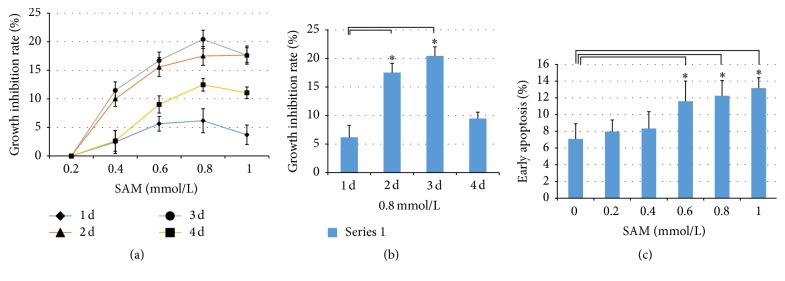
Effects of DNA methylation status of miR-23a-27a-24-2 cluster promoter CG-rich region on proliferation and apoptosis. (a) Inhibition of different concentrations of SAM on Hep2 cell growth. (b) Inhibition analysis of 0.8 mmol/L SAM on Hep2 cell growth. (c) Early apoptosis analysis of different concentrations of SAM on Hep2 cells on the third day. *∗* indicates *P* < 0.05.

To identify whether methylation status of the CpG-rich region affects Hep2 cell functions, we detected Hep2 cell proliferation and apoptosis by MTT and flow cytometry methods, respectively. MTT results showed a decreased viability tendency with the increase of concentration and treatment duration of SAM compared to the controls (Figure 2(a)). Moreover, Hep2 cell proliferation reached a peak at 0.8 mmol/L of SAM treatment on either day and showed significant differences at 0.8 mmol/L on the second and third day (Figure 2(b)). On the contrary, SAM-treated Hep2 cells displayed an increased trend in early apoptosis with an increase in SAM concentration on the third day and began to reveal a significant difference at 0.6 mmol/L compared to the controls (Figure 2(c)). We speculate that the CG-rich region hypomethylation partly regulates the Hep2 cell proliferation and early apoptosis.

#### 3.1.4. SP1 Sites within the CG-Rich Region Are Important in Regulation of miR-23a-27a-24-2 Cluster

**Figure 3:**
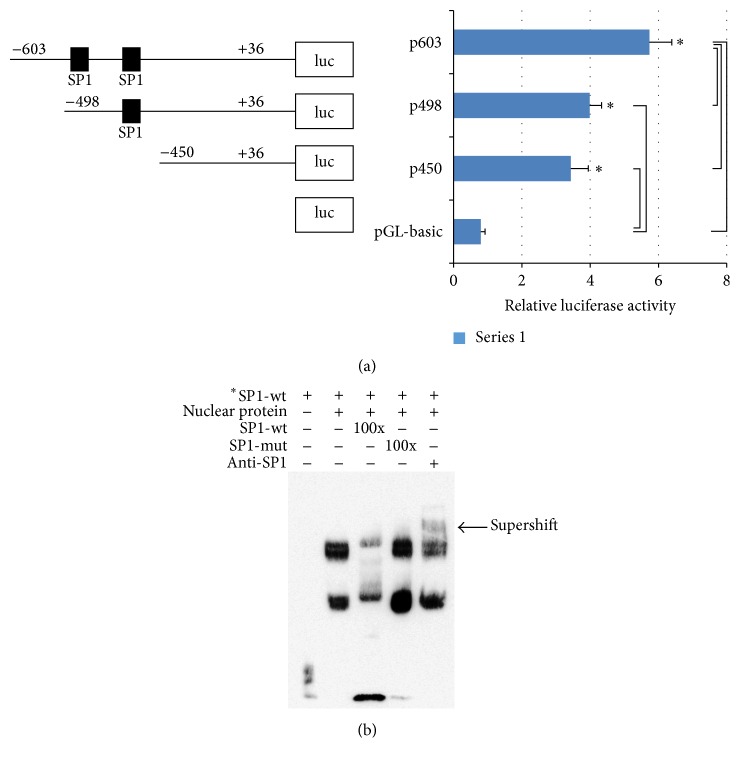
Activities of miR-23a-27a-24-2 cluster promote and binding of SP1 to the cluster CG-rich region. (a) Relative luciferases of different constructs in miR-23a-27a-24-2 cluster are promoted. (b) Binding of SP1 to the cluster CG-rich region in vitro. *∗* indicates *P* < 0.05.

Luciferase reporter assay result indicated that construct overlapping two SP1 sites had strongest luciferase activity and even that harbouring one SP1 site showed significantly higher luciferase ability compared to the controls (Figure 3(a)), implying that the SP1 sites probably regulate miR-23a-27a-24-2 cluster expression. EMSA result displayed that SP1-labled wild type probe had strong binding ability to nuclear proteins compared to the probe-free group. By addition of anti-SP1 antibody, a shifting band was detected. SP1-unlabeled probe reduced the binding and SP1-mutant probe had no effect on the binding (Figure 3(b)). The findings confirmed that both SP1 sites are import cis-acting elements in miR-23a-27a-24-2 cluster regulation.

### 3.2. Discussion

It is well-known that transcription factors regulate target gene expression via binding cis-acting elements [24]. As a basal transcription factor, SP1 plays a critical role in regulation of so-called housekeeping genes especially in the absence of TATA box [25].

miR-23a-27a-24-2 cluster promoter is located at the region −603 bp~+38 bp that lacks the known common promoter element TATA box [26, 27]. In the study, we first found a CG-rich region with six CpG dinucleotides covering two SP1 sites. Compared to HEK-293 cells, we discovered that the region is hypomethylated where both SP1 sites are demethylated in Hep2 cells. Meanwhile, all members of the cluster are significantly upregulated in Hep2 cells compared to HEK-293 cells, suggesting that hypomethylation especially demethylation of both SP1 sites in the region might contribute to overexpression of the cluster. SAM, S-adenosyl-L-methionine, is a very useful methyl donor in epigenetic mechanism study [28]. In the present study, we also found most cytosines in the CG-rich region are methylated in Hep2 cells after being treated by SAM. All three miRNAs of miR-23a-27a-24-2 cluster are significantly downregulated in SAM-treated Hep2 cells compared to the controls, further indicating that abnormal methylation in the CG-rich region regulates the cluster expression.

Recently, miR-23a and 27a are reported in an aberrant methylation status in several studies. Similar to our results, miR-23a overexpression is speculated to be associated with its hypomethylation in leukemic cells [29]. In hepatocellular carcinoma, hypomethylation is considered to be responsible for upregulation of miR-23a and miR-27a [13]. On the contrary, the miR-23a gene promoter region was found to be hypermethylated, leading to downregulation of miR-23a in osteosarcoma cells [21]. However, these results do not tell us the detailed information on alteration of methylation status and molecular mechanism of which methylation-related sites could affect cancer cell function.

It is also well-known that miRNAs participate in regulation of cancer cell proliferation, apoptosis, differentiation, migration, and metastasis via different targets [30–33], respectively. At present, lots of targets of miR-23a-27a-24-2 cluster have been identified such as GJA1, BCL2, CDC27, 14-3-3*θ*, NAIF1, and SOX7 [34–39]. In our previous study, we found that miR-23a and miR-27a overexpression in Hep2 cells promotes cancer cell proliferation and represses apoptosis by targeting APAF-1 and PLK2 [22, 23], respectively.

In the present study, SAM-treated Hep2 cells showed significantly lower level of proliferation and higher level of apoptosis than controls, indicating that SAM increases proliferation and decreases apoptosis in laryngeal cancer cells. SAM-induced proliferation prevention and apoptosis activation have been found in other studies. For example, SAM inhibits osteosarcoma and colorectal cancer cell proliferation [40, 41]. SAM causes apoptosis in normal liver L-02 cells and undifferentiated pheochromocytoma PC12 cells [42, 43]. Our EMSA result indicates that SP1 is an important* trans*-acting factor of miR-23a-27a-24-2 cluster and mutant SP1 reduces the banding ability of SP1 to the cluster promoter. Similar to the SP1 variation, we suggest that methylation status of SP1 sites may interfere with the binding of SP1 to miR-23a-27a-24-2 cluster. It has been shown that CpG methylation in the promoter region of BLU could prevent itself from binding to SP1 [44].

## 4. Conclusion

We conclude that demethylated SP1 sites in CG-rich region of miR-23a-27a-24-2 cluster promoter result in the cluster overexpression, leading to proliferation promotion and apoptosis inhibition probably via targeting the related targets such as APAF-1 and PLK2 in laryngeal cancer cells. These provide us with an important basis in our future work on miR-23a-27a-24-2 cluster promoter methylation in human cancer tissues and its clinical significance in tumorigenesis.

## Figures and Tables

**Table 1:** Primer sequences of miR-23a/27a/24-2 cluster used in the study.

Primer name	Sequence
miR-23a	F: 5′-ATCAC ATTCGCAGGGATTTCC-3′
	RTQ-UNIr:
	5′-CGAATTCTAGACGTCGAGCGAGCGGA CATGCGTGCGTAGTTAACGTTGGTACCGACGTCGGATCCACTAGTCC (T)-3′

miR-27a	F: 5′-TTCACAGTGCGTAAGTTCCCG-3′
	RTQ-UNIr:
	5′-CGAATTCTAGACGTCGAGCGAGCGGA CATGCGTGCGTAGTTAACGTTGGTACCGACGTCGGATCCACTAGTCC (T)-3′

miR-24-2	F: 5′-TGCGTCAGTTCACGAGGAACAG-3′
	RTQ-UNIr:
	5′-CGAATTCTAGACGTCGAGCGAGCGGA CATGCGTGCGTAGTTAACGTTGGTACCGACGTCGGATCCACTAGTCC (T)-3′

U6	F: 5′-CTCCGTTCGCGACGACA-3′
	R: 5′-AACCGTTCACGAATTTCGGT-3′
